# NUTRIC and Modified NUTRIC are Accurate Predictors of Outcome in End-Stage Liver Disease: A Validation in Critically Ill Patients with Liver Cirrhosis

**DOI:** 10.3390/nu12072134

**Published:** 2020-07-17

**Authors:** Ulrich Mayr, Julia Pfau, Marina Lukas, Ulrike Bauer, Alexander Herner, Sebastian Rasch, Roland M. Schmid, Wolfgang Huber, Tobias Lahmer, Gonzalo Batres-Baires

**Affiliations:** Klinik und Poliklinik für Innere Medizin II, Klinikum rechts der Isar, Technische Universität München, Ismaninger Strasse 22, D-81675 München, Germany; info.juliapfau@gmail.com (J.P.); marina.lukas@mri.tum.de (M.L.); ulrike.bauer@mri.tum.de (U.B.); alexander.herner@mri.tum.de (A.H.); sebastian.rasch@mri.tum.de (S.R.); rolandM.schmid@mri.tum.de (R.M.S.); tobias.lahmer@mri.tum.de (T.L.); batresbaires@web.de (G.B.-B.)

**Keywords:** liver cirrhosis, intensive care unit, NUTRIC, modified NUTRIC, nutritional risk, mortality risk

## Abstract

Malnutrition in critically ill patients with cirrhosis is a frequent but often overlooked complication with high prognostic relevance. The Nutrition Risk in Critically ill (NUTRIC) score and its modified variant (mNUTRIC) were established to assess the nutrition risk of intensive care unit patients. Considering the high mortality of cirrhosis in critically ill patients, this study aims to evaluate the discriminative ability of NUTRIC and mNUTRIC to predict outcome. We performed a retro-prospective evaluation in 150 Caucasian cirrhotic patients admitted to our ICU. Comparative prognostic analyses between NUTRIC and mNUTRIC were assessed in 114 patients. On ICU admission, a large proportion of 65% were classified as high NUTRIC (6–10) and 75% were categorized as high mNUTRIC (5–9). High nutritional risk was linked to disease severity and poor outcome. NUTRIC was moderately superior to mNUTRIC in prediction of 28-day mortality (area under curve 0.806 vs. 0.788) as well as 3-month mortality (area under curve 0.839 vs. 0.819). We found a significant association of NUTRIC and mNUTRIC with MELD, CHILD, renal function, interleukin 6 and albumin, but not with body mass index. NUTRIC and mNUTRIC are characterized by high prognostic accuracy in critically ill patients with cirrhosis. NUTRIC revealed a moderate advantage in prognostic ability compared to mNUTRIC.

## 1. Introduction

Liver cirrhosis causes predispositions to serious complications due to the progressive impairment of hepatocellular function and portal hypertension [1,2,3]. Acute decompensation and successive organ failure frequently lead to vital threats following transfer to an intensive care unit (ICU) [4]. End-stage liver disease is a challenge to intensivists as intraabdominal hypertension, bleeding episodes, hepato-renal syndrome or infectious complications imply poor prognosis [5,6,7,8,9]. Mortality rates in ICU patients with cirrhosis are dramatically high [10,11,12,13,14]. As a consequence, the early and accurate estimation of prognosis is of crucial relevance in critically ill patients with cirrhosis [15].

In recent years, malnutrition in patients with liver disease has drawn increasing attention to clinical research. Development of malnutrition is multifactorial depending on decreased oral intake, malabsorption, disturbed metabolism and an often catabolic or chronic inflammatory state resulting in an increased resting metabolic rate [16,17,18,19,20]. Malnutrition in cirrhosis is an important predictor of mortality and associated with decreased pre- and posttransplant survival [21,22]. However, consensus about the approach to nutritional status in cirrhosis is still lacking and assessment may be impaired by ascites or edema [23]. Despite its prognostic role, the nutritional status is often overlooked in contrast to more notable cirrhotic complications [24].

Malnutrition is highly prevalent in ICU patients and a risk factor for poor outcome [25,26]. However, common methods to assess nutritional status are heterogenous, time-consuming or applicable only to a limited extent in ICU [27,28]. Malnutrition in critically ill patients is not only referable to chronic and acute starvation, but also influenced by inflammatory mechanisms leading to stress responses and catabolic metabolism [29]. Moreover, patients at a higher risk of malnutrition are more likely to benefit from early nutrition therapy [30]. Therefore, accurate tools should help to discriminate nutritional risk and identify individuals which are more likely to profit from intensified nutritional support. The Nutrition Risk in Critically ill (NUTRIC) score was developed as a rapid and smart risk assessment in a heterogenous population of ICU patients. The score contains the variables age, co-morbidities, days from hospital admission to ICU transfer, Acute Physiology and Chronic Health Evaluation II (APACHE II), Sequential Organ Failure Assessment (SOFA) and interleukin 6 (IL6) [29]. Applicability in clinical routine was further expanded by waiving IL6 in the modified variant mNUTRIC [30]. According to earlier results, both NUTRIC and mNUTRIC revealed prognostic accuracy especially in ICU patients at a high risk for a worse outcome [29,31].

Concerning critically ill patients with cirrhosis, reliable data about nutritional risk assessment are rare. Furthermore, comparative evaluation of NUTRIC and mNUTRIC in this specific subgroup of ICU patients is lacking so far. In consideration of the high mortality of end-stage liver disease, predictors of mortality are of outstanding relevance. This study aims to assess the prognostic accuracy of NUTRIC compared to mNUTRIC in a challenging population of ICU patients with cirrhosis.

## 2. Materials and Methods

### 2.1. Study Design

This retro-prospective study was approved by the institutional review board (Ethikkommission Technische Universität München; Fakultät für Medizin; Project number 431/19 S-SR). Due to its retrospective nature informed consent is not feasible. From January 2016 to January 2020, we screened all patients with liver cirrhosis admitted to our 10-bed, university hospital medical intensive care unit. Treatment followed current standards in our ICU irrespective of this study and nutritional support was based on guidelines for clinical nutrition in liver disease [30,32]. Diagnosis of cirrhosis was established on the following features: Previous medical reports suggesting end-stage liver disease (i.e., episodes of ascites, variceal bleeding or hepatic encephalopathy), imaging methods with typical morphological criteria or histological characteristics of cirrhosis, laboratory disorders signaling impaired liver synthesis in presence of risk factors for cirrhosis.

We excluded all patients with incomplete knowledge about individual or laboratory data to assess clinical scores evaluated in this study (*n* = 13). All patients who underwent liver transplantation during observation period (*n* = 3) and cirrhotics with hepatocellular carcinoma (*n* = 3) were excluded due to obvious influences on outcome. Furthermore, we excluded patients lost during the observation period (*n* = 3) and patients re-admitted after prior ICU treatment in our hospital (*n* = 7).

We enrolled a total of 150 Caucasian patients with liver cirrhosis admitted to our ICU. Baseline analysis of interleukin 6 (IL6) was not available in blood samples of 36 patients to calculate original NUTRIC score. Finally, comparative prognostic analyses of NUTRIC and modified NUTRIC (mNUTRIC) were performed in 114 patients.

### 2.2. Blood Sampling and Laboratory Analyses

All blood samples were acquired corresponding to current standard in our ICU either from central venous catheters or arterial lines. Laboratory results were included in the calculation of disease severity scores like the APACHE II or SOFA scores. Parameters of hepatic function were used for staging of cirrhosis in terms of CHILD and Model of end-stage liver disease (MELD) scores.

IL6 is an essential feature in assessment of NUTRIC score. Laboratory analysis was done by using an electrochemiluminescence immunoassay (ECLIA) with a detection limit of 1.5 pg/mL (Cobas 8000^®^, Roche, Basel, Switzerland). Baseline measurement of IL6 is done routinely in patients with liver cirrhosis admitted to our ICU. All laboratory tests were realized by the department of clinical laboratory chemistry of our university hospital.

### 2.3. Calculation of NUTRIC and mNUTRIC

We used laboratory data and features of individual health state for assessment of NUTRIC and mNUTRIC. As described previously, calculation includes demographics (age), number of co-morbidities, days of hospital admission to ICU transfer, APACHE II score, SOFA score and IL6 during the first 24 h after admission to ICU [29]. The formula utilized for classification of NUTRIC is shown in Table 1. Analogously, mNUTRIC was calculated as a modification including all variables of original NUTRIC with exception of IL6 [31]. According to earlier results, we further distinguished between low NUTRIC (0–5) and high NUTRIC (6–10) as well as low mNUTRIC (0–4) and high mNUTRIC (5–9) [29,31].

### 2.4. Data Collection

All clinical, laboratory and individual parameters for the calculation of APACHE II, SOFA, MELD, CHILD, NUTRIC and mNUTRIC scores were recorded from the day of admission to ICU. Patients were followed up until death or observed for 3 months in order to conduct survival analyses 28 days and 3 months after ICU admission.

### 2.5. Statistical Analysis and Primary Endpoint

Continuous variables were depicted as median and interquartile range (IQR), due to the fact that variables were predominantly not normally distributed. Categorical variables were outlined as percentages. To compare patient cohorts we used nonparametric, two-tailed Mann–Whitney test. Receiver–operating–characteristic curves (ROC) were used to express the ability of different variables for prediction of 28-day and 3-month mortalities via area under curve (AUC). Appropriate cut-offs were identified by highest combined sensitivity and specificity using Youden’s index. Positive predictive value (PPV) and negative predictive value (NPV) to predict mortality were calculated for NUTRIC and mNUTRIC. Univariate analyses were used to evaluate associations of various baseline parameters with mortality. Independent predictors of mortality were assessed via multivariate logistic regressions. Survival analyses were performed according to the Kaplan–Meier method, whereby all deaths were recorded as events. We used Log-rank (Mantel–Cox) test for the comparison of survival curves. Associations of variables with mortality risk were calculated as hazard ratio (HR) by Mantel–Haenszel. Correlations were calculated by using Spearman’s coefficient r_s_ and linear regressions using the coefficient R^2^. In each case, significance was assumed at a *p*-value < 0.05. All analyses and graphs were generated using GraphPad Prism 8.0 (GraphPad Software, La Jolla, CA, USA).

## 3. Results

### 3.1. Patients’ Characteristics and Laboratory Analyses

A total of 150 critically ill patients with liver cirrhosis were included. As the baseline analysis of IL6 was missing in 36 patients, we performed a comparative evaluation of NUTRIC and mNUTRIC in a subgroup of 114 patients. The etiology of cirrhosis was predominantly alcoholic–toxic. The corresponding baseline characteristics on admission to ICU are presented in Table 2.

A comparison of characteristics between patients ‘Included’ (*n* = 114) and those ‘Not included’ (*n* = 36) in further analyses is listed in Table S1.

### 3.2. Mortality Risk Depending on NUTRIC and mNUTRIC

**Figure 1:**
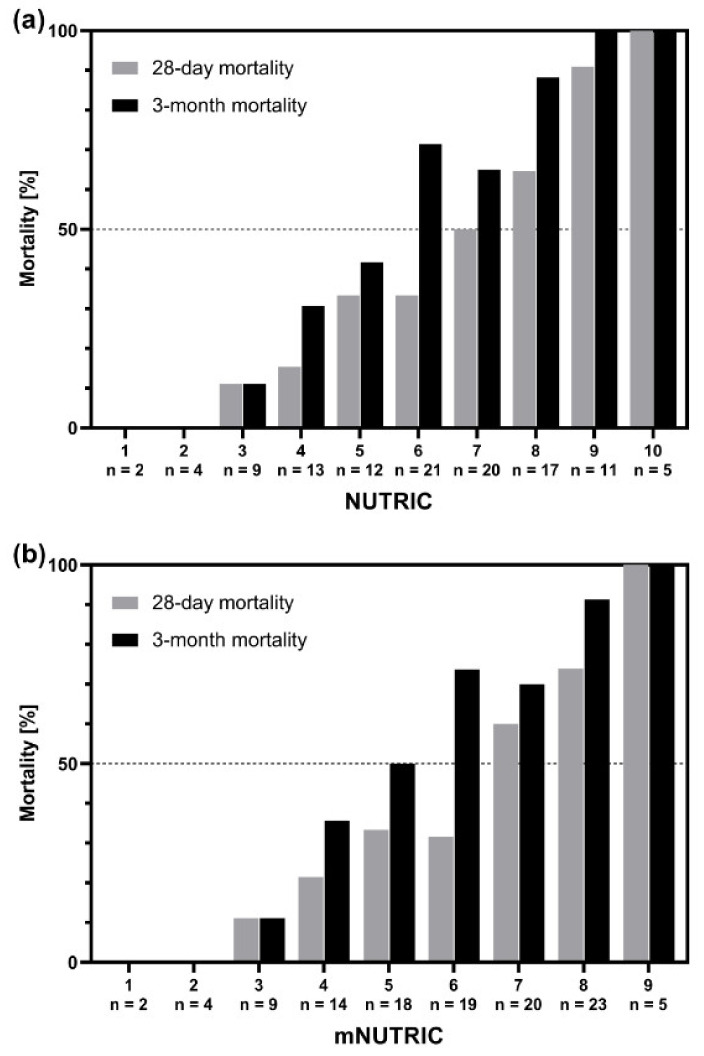
Mortality rates 28 days and 3 months after admission to ICU in dependence of baseline (**a**) NUTRIC and (**b**) mNUTRIC.

Mortality rates in a total of 114 cirrhotic patients according to NUTRIC and mNUTRIC are illustrated in Figure 1: Worsening scores were associated with dramatically decreased survival proportions 28 days and 3 months after admission to ICU.

Correspondingly, survival analyses by Kaplan–Meier showed marked differences depending on admission levels. Patients with high NUTRIC scores between 6–10 revealed a higher risk for short-term mortality 28 days after admission to ICU than cirrhotics with NUTRIC scores between 0–5 (HR 3.31, 95% CI = 1.87–5.86, *p* < 0.001). In a similar way, higher NUTRIC scores were associated with significantly higher mortality risk 3 months after ICU admission (HR 3.86, 95% CI = 2.38–6.27, *p* < 0.001).

**Figure 2:**
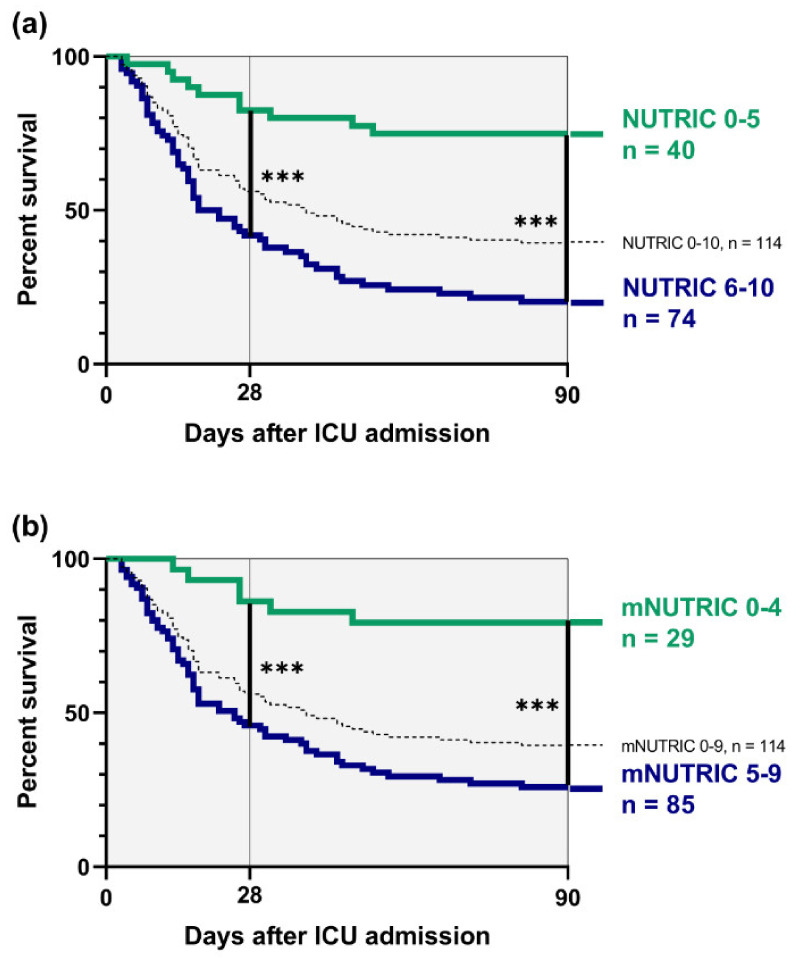
Survival analyses depending on baseline scores (**a**) Low NUTRIC 0–5 (*n* = 40) vs. high NUTRIC 6–10 (*n* = 74) (**b**) Low mNUTRIC 0–4 (*n* = 25) vs. high NUTRIC 5–9 (*n* = 85); *** = *p* < 0.001.

Cirrhotic patients with high mNUTRIC scores between 5–9 had a significantly increased mortality risk compared to lower mNUTRIC scores between 0–4, concerning both 28-day mortality (HR 3.10, 95% CI = 1.69–5.68, *p* < 0.001) as well as 3-month mortality (HR 3.33, 95% CI = 2.01–5.50, *p* < 0.001). Survival curves showed an almost congruent course for NUTRIC (Figure 2a) and mNUTRIC (Figure 2b).

Furthermore, a comparison of patients with low NUTRIC or low mNUTRIC to high NUTRIC or high mNUTRIC, respectively, is shown in Table 3; A substantial proportion of 65% was classified as high NUTRIC and a fraction of 75% as high mNUTRIC. Higher risk scores were associated with increased disease severity, length of ICU stay and mortality: High NUTRIC and mNUTRIC revealed not only worse APACHE II and SOFA, but also more advanced stages of cirrhosis in terms of MELD and CHILD scores, higher IL6 as well as worse renal function.

### 3.3. Patients with Discrepancy between NUTRIC and mNUTRIC

Discrepancy between NUTRIC and mNUTRIC was apparent in 34 out of 114 patients (30%) due to admission levels of IL6 ≥ 400 pg/mL. In this subgroup we found extremely high parameters of disease severity with a median APACHE II of 28 (22–32), SOFA of 14 (9–16), MELD of 32 (25–36) and CHILD score of 12 (11–14). Cumulative mortality rates reached 74% after 28 days and 91% after 3 months. This subgroup revealed a median NUTRIC of 8 (7–9) and mNUTRIC of 7 (6–8). Infectious diseases and sepsis were the predominant fraction of admission diagnoses (59%) and causes of death (68%).

### 3.4. Prognostic Accuracy of NUTRIC and mNUTRIC

**Figure 3:**
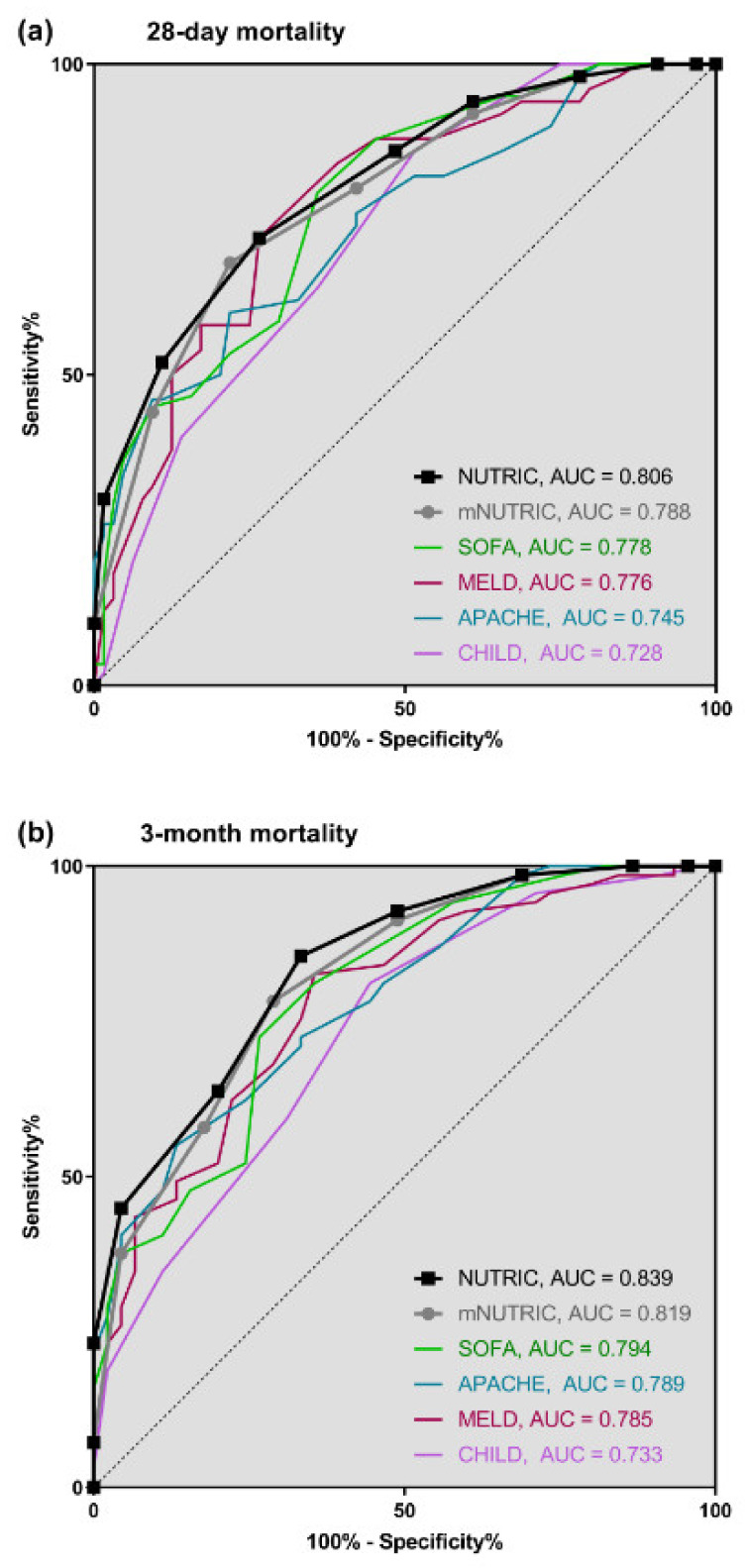
Prognostic accuracy of NUTRIC and mNUTRIC to predict outcome in comparison to APACHE II, SOFA, MELD and CHILD: (**a**) 28 days (**b**) 3 months after admission to ICU.

For primary outcome analysis, we used ROC-curves to compare prediction of patient´s outcome; As depicted in Figure 3, NUTRIC and mNUTRIC revealed a sufficient prognostic potential in a population of 114 critically ill cirrhotics. Concerning 28-day mortality, the prognostic value of NUTRIC (AUC = 0.806) was slightly higher than that of mNUTRIC (AUC = 0.788). NUTRIC had a PPV of 67.9% and an NPV of 77.1% in the prediction of 28-day mortality, compared to mNUTRIC with a PPV of 70.8% and an NVP of 75.8%. The highest combined sensitivity and specificity was found with a cut-off of ≥ 7, respectively. In relation to common clinical scores, NUTRIC and mNUTRIC performed better than APACHE II (AUC = 0.745), SOFA (AUC = 0.778), MELD (AUC = 0.776) and CHILD scores (AUC = 0.728). Figure 3a demonstrates the comparison of ROC-curves. By contrast, body mass index (BMI) (AUC = 0.524) and albumin (AUC = 0.539) were not helpful to predict 28-day mortality.

Prediction of mortality 3 months after ICU admission is shown in Figure 3b: Prognostic accuracy of NUTRIC (AUC = 0.839) was higher than that of mNUTRIC (AUC = 0.819). Our analyses for NUTRIC resulted in a PPV of 79.7% and a NPV of 75.0%, in comparison to mNUTRIC with a PPV of 80.6% and a NPV of 68.1%. NUTRIC revealed a sensitivity of 85.5% and a specificity of 66.7% compared to mNUTRIC with a sensitivity of 78.3% and a specificity of 71.1% (cut-off ≥ 6, respectively). The ability to predict unfavorable outcome was better in comparison to APACHE II (AUC = 0.789), SOFA (AUC = 0.794), MELD (AUC = 0.785) and CHILD scores (AUC = 0.733). Analogously to the results mentioned above, BMI (AUC = 0.518) and albumin (AUC = 0.584) had no prognostic relevance.

In addition, we performed univariate and multivariate analyses to identify independent predictors of mortality. Univariate analyses in 16 baseline parameters is shown in Table S1: Significant associations of variables with 28-day mortality and 3-month mortality, respectively, are marked in bold.

Multivariate analyses were created separately for NUTRIC and mNUTRIC, as illustrated in Table S2. Model 1 was built by including all variables significantly linked to outcome in corresponding univariate analyses. To avoid collinearities, we further excluded parameters that were components of nutritional risk assessment (APACHE II, SOFA, co-morbidities) in Model 2. In each model, NUTRIC was an independent risk factor for 28-day and 3-month mortalities, respectively

### 3.5. Correlation Analyses

Correlation analyses of NUTRIC and mNUTRIC with various parameters are listed in Table 4; According to Spearman, we found a significant correlation of NUTRIC and mNUTRIC with MELD (*p* < 0.001 vs. *p* < 0.001) and CHILD (*p* < 0.001 vs. *p* < 0.001). Furthermore, both scores were associated with IL6 (*p* < 0.001 vs. *p* < 0.001). Concerning simple parameters of malnutrition, our analyses revealed a statistically significant inverse association of NUTRIC and mNUTRIC with albumin (*p* = 0.008 vs. *p* = 0.013). As opposed to this, we found no significant correlation of NUTRIC or mNUTRIC with BMI (*p* = 0.479 vs. *p* = 0.422) on admission to ICU.

Concerning associations of nutritional risk with renal function, we excluded two patients with pre-existing hemodialysis due to chronic renal insufficiency. In 112 consecutive patients, we found a highly significant correlation of baseline creatinine with NUTRIC (r_s_ = 0.500, R^2^ = 0.167, *p* < 0.001) and mNUTRIC (r_s_ = 0.517, R^2^ = 0.175, *p* < 0.001) on admission to ICU. Moreover, both scores performed well in prediction of need for hemodialysis during ICU stay of cirrhotic patients (Figure S1): NUTRIC had a sensitivity of 68.2% and a specificity of 84.8% (cut-off ≥ 7, AUC = 0.844) compared to mNUTRIC with a sensitivity of 63.6% and a specificity of 89.1% (cut-off ≥ 7, AUC = 0.841).

## 4. Discussion

This study primarily illustrates the prognostic accuracy of NUTRIC in comparison to modified NUTRIC (mNUTRIC) in critically ill patients with liver cirrhosis.

More precisely, NUTRIC revealed a slightly higher prognostic validity than mNUTRIC to predict mortality 28 days as well as 3 months after ICU admission. In this specific population of critically ill patients with liver cirrhosis, both tools performed better in outcome prediction than most commonly used disease severity scores APACHE II, SOFA, MELD or CHILD. Considering the dramatically high mortality rates of advanced liver disease with often tremendous use of critical care resources, accurate prognostic tools are of crucial relevance for individualized treatment of cirrhosis.

The moderate advantage in prognostic ability of NUTRIC is obviously referable to the incorporation of interleukin 6 (IL6) as all other components are identical to mNUTRIC [29,31]. In our population of ICU patients with cirrhosis, about one third had varying levels for NUTRIC and mNUTRIC with IL6 ≥ 400 pg/mL. Correspondingly, sepsis and infectious diseases were the main part of admission diagnoses and causes of death in this subgroup. Moreover, cirrhotics with IL6 ≥ 400 pg/mL suffered from particularly severe morbidity and mortality. Various previous studies addressed the high incidence of bacterial infections and septic multi-organ failure referable to a complex syndrome of cirrhosis associated immune dysfunction (CAID) [33,34,35]. Associations of proinflammatory markers like CRP, PCT or IL6 with increased mortality are well-characterized in cirrhotics [36,37,38,39]. Inflammatory responses play an important role in increased resting energy expenditure and catabolic metabolism [18,40]. Taken together, the additional effort of measuring IL6 incorporated in NUTRIC might offer valuable additional information regarding nutritional risk assessment as well as outcome prediction in ICU patients with liver cirrhosis.

Therefore, our results are to some extent different from previous studies. A large study in 482 patients with sepsis described similar prognostic accuracy between NUTRIC and mNUTRIC for 28-day mortality [41]. The authors concluded that incorporation of IL6—which is often not routinely assessed—may be superfluous as part of nutritional risk assessment. In contrast to our study, they found an inferiority of NUTRIC and mNUTRIC to APACHE II in prediction of mortality. However, comparison of both studies is difficult as our cirrhotic population had a large proportion of patients within the high-risk stages with correspondingly dramatically high mortality rates.

Another finding deviating from previous evaluations is about the appropriate cut-off for NUTRIC and mNUTRIC to stratify between low and high risk. According to Youden’s index, we found a suitable cut-off ≥ 6 for both NUTRIC and mNUTRIC to predict 3-month mortality. Analogously, ROC-analyses revealed a cut-off ≥ 7 to predict 28-day mortality. By contrast, Jeong et al. found the best cut-off ≥ 6 for mNUTRIC with a sensitivity of 75% and a specificity of 65% to predict 28-day mortality [41]. Another study described an appropriate cut-off ≥ 5 for mNUTRIC in a mixed ICU population of 401 patients [42]. De Vries et al. found the best discriminative ability with a mNUTRIC cut-off > 4 for 28-day mortality in 475 mechanically ventilated patients [43]. A large study in 1143 patients also used a cut-off ≥ 5 for mNUTRIC to predict the outcome 28 days after ICU admission [44]. Despite these former findings, data evaluating the appropriate cut-off for NUTRIC in specific cohorts of ICU patients are rare. According to the original evaluation in 597 heterogenous ICU patients, a cut-off ≥ 6 for NUTRIC was proposed to characterize high nutritional risk [29]. However, disease severity in terms of APACHE II, SOFA and outcome are varying between different ICU populations. Thus, higher levels of appropriate cut-offs could be due to pronounced illness in our population of ICU patients with cirrhosis. Further investigations are required to define the best cut-offs for NUTRIC and mNUTRIC in specific populations.

Nevertheless, our study is the first comparing the prognostic ability of NUTRIC and mNUTRIC in ICU patients with cirrhosis. Recently, a retrospective study by Tsai et al. evaluated the nutritional risk assessment via mNUTRIC in 120 patients with acute gastroesophageal variceal bleeding [45]. They described an excellent discriminating power to predict 6-week mortality (AUC 0.859) and mNUTRIC was associated with increased morbidity concerning MELD and CHILD. Moreover, the authors revealed a correlation of nutritional risk with CRP confirming the hypothesis that inflammatory response is a key component of malnutrition and poor outcome in cirrhosis [16,18]. Analogously, both NUTRIC and mNUTRIC were associated with IL6 in our study. Therefore, the question might not be whether IL6 is superfluous but perhaps interchangeable with more widely available proinflammatory markers instead.

Finally, higher levels of NUTRIC and mNUTRIC were significantly associated with advanced hepatic impairment and increased mortality in our study. In addition, both scores were associated with renal function and performed well to predict need for hemodialysis during an ICU stay. Concerning simple parameters of malnutrition, we found significant correlations with albumin, but not with BMI. This result is in line with the original study by Heyland et al. stating that BMI did not have a significant relation with 28-day mortality [29]. Their conceptual model was designed to link starvation and inflammation to patient´s outcome [46], but spares the traditional parameters BMI, physical assessment, weight loss or oral intake [28,29]. NUTRIC was developed as an easily applicable and accurate scoring system in an ICU setting for the early identification of patients most likely to benefit from intensive nutritional support [29]. However, most available studies addressed associations with prognosis, but had no interventional design. Rahman et al. noticed a positive association of nutritional adequacy with survival rates, especially in patients with high mNUTRIC scores [31]. Another study by Compher et al. revealed a promising reduction of mortality in high-risk patients by an increase in protein/energy intake [47]. Thus far, evaluations in cirrhotics focusing on the effects of nutrition therapy in case of high risk are lacking. The large proportion of patients with high nutritional risk and poor prognosis in our study emphasizes the challenging character of advanced cirrhosis to intensivists. The present results underline the need for further studies addressing individualized nutritional approach based on NUTRIC and mNUTRIC.

## 5. Limitations

Although the results are conclusive with high levels of statistical significance, this study has several limitations: It is a single-center study performed exclusively in an ICU setting with a limited number of patients with cirrhosis. There is no comparison to other ICU populations. The major etiology of cirrhosis was alcoholic–toxic in our Caucasian cohort of critically ill patients, whereas viral-related cirrhosis still plays an important role worldwide. The study focuses on baseline assessment of NUTRIC and mNUTRIC, whereas further evaluation in the course of ICU treatment is not available. The primary outcome analysis is restricted to 28-day and 3-month mortalities, whereas ICU mortality is not taken into account. Moreover, this study has no interventional design. Effects and adequacy of nutritional therapy on outcome or length of ICU stay were not assessed. This limitation reemphasizes the need for prospective studies based on NUTRIC or mNUTRIC.

## 6. Conclusions

The present study is the first one comparing the prognostic relevance of NUTRIC and mNUTRIC in critically ill patients with liver cirrhosis. It demonstrates an association of nutritional risk assessment not only with outcome, but also with disease severity, inflammatory state and renal function. In a particularly ill population of ICU patients with cirrhosis, NUTRIC had a moderate prognostic advantage in comparison to mNUTRIC.

## Figures and Tables

**Table 1:** The scoring system for classification of NUTRIC and mNUTRIC [29,31].

Variables	Scoring System (Points)			
Included in NUTRIC	0	1	2	3
Ages, years	<50	50–74	≥75	
Co-morbidities	0–1	≥2		
Days from hospital to ICU	0	≥1		
APACHE	<15	15–19	20–27	≥28
SOFA	<6	6–9	≥10	
Interleukin 6, pg/mL	<400	≥400		
Low NUTRIC	0–5 Points			
High NUTRIC	6–10 Points			
**Modified NUTRIC**	(without Interleukin 6)			
Low mNUTRIC	0–4 Points			
High mNUTRIC	5–9 Points			

ICU: Intensive care unit; APACHE: Acute physiology and chronic health evaluation; SOFA: Sequential organ failure assessment.

**Table 2:** Patients’ characteristics.

Male sex, n/total (%)	**72/114 (63%)**
Age, years	61 (52–67)
Body weight, kg	75 (68–85)
Body height, cm	175 (167–180)
BMI, kg/m2	24.8 (22.5–27.7)
APACHE II	22 (17–28)
SOFA	10 (8–13)
MELD	26 (22–32)
Child-Pugh	11 (10–13)
Child C, n/total (%)	98/114 (86%)
Etiology of cirrhosis, n/total (%)	Alcoholic 78/114 (68%)
	Viral 9/114 (8%)
	Autoimmune 5/114 (4%)
	Cryptogenic/NAFLD 22/114 (20%)
Admission diagnoses, n/total (%)	Sepsis/Pneumonia 50/114 (44%)
	Acute kidney failure/HRS 24/114 (21%)
	Gastrointestinal bleeding 20/114 (18%)
	Encephalopathy/delirium 20/114 (17%)
Length of ICU stay, days	13 (6–22)
28-day mortality, n/total (%)	50/114 (44%)
3-month mortality, n/total (%)	69/114 (61%)
Clinical cause of death, n/total (%)	Sepsis, Pneumonia 41/69 (61%)
	Cardiocirculatory failure 13/69 (19%)
	Gastrointestinal bleeding 11/69 (16%)
	Central-nervous limitations 3/69 (4%)
Baseline creatinine, mg/dL	1.8 (1.2–2.7)
Dialysis before ICU, n/total (%)	2/114 (1.8%)
Dialysis during ICU, n/total (%)	66/112 (59%)

BMI: Body mass index; APACHE: Acute physiology and chronic health evaluation; SOFA: Sequential organ failure assessment; MELD: Model of end-stage liver disease; NAFLD: Non-alcoholic fatty liver disease; HRS: Hepato-renal syndrome; ICU: Intensive care unit.

**Table 3:** Characteristics of patients with low NUTRIC (0–5) and mNUTRIC (0–4) to patients with high NUTRIC (6–10) and mNUTRIC (5–9), respectively.

	NUTRIC, *n* = 114			mNUTRIC, *n* = 114		
	Low NUTRIC 0–5 *n* = 40	High NUTRIC 6–10 *n* = 74	*p*-Value	Low mNUTRIC 0–4 *n* = 29	High mNUTRIC 5–9 *n* = 85	*p*-Value
Age, years	55 (43–61)	64 (55–68)	<0.001	54 (44–60)	63 (55–68)	<0.001
Height, cm	175 (167–177)	175 (167–180)	0.449	174 (167–177)	175 (168–180)	0.155
Weight, kg	73 (65–81)	76 (69–85)	0.206	71 (64–79)	76 (69–85)	0.056
BMI, kg/m^2^	23.8 (21.8–27.4)	25.2 (22.7–28.0)	0.200	23.7 (22.0–26.9)	25.2 (22.5–28.3)	0.134
Albumin, g/dL	3.3 (2.7–3.9)	3.0 (2.5–3.5)	0.048	3.4 (2.9–3.9)	3.0 (2.5–3.6)	0.050
Co-morbidities	2 (2–3)	3 (3–4)	<0.001	2 (2–3)	3 (3–4)	<0.001
Days from hospital to ICU	1 (0–3)	3 (1–5)	0.006	1 (0–3)	3 (1–5)	0.029
Interleukin 6, pg/mL	64 (32–160)	246 (57–895)	<0.001	40 (23–127)	204 (60–694)	<0.001
Creatinine, mg/dL	1.3 (1.0–1.9)	2.2 (1.6–3.2)	<0.001	1.1 (0.8–1.7)	2.1 (1.5–3.1)	<0.001
APACHE	17 (14–18)	25 (22–29)	<0.001	15 (12–17)	25 (22–28)	<0.001
SOFA	6 (5–8)	12 (10–15)	<0.001	6 (4–7)	11 (9–15)	<0.001
MELD	23 (20–28)	28 (24–34)	<0.001	22 (20–26)	27 (23–34)	<0.001
CHILD	9 (10–12)	12 (11–13)	<0.001	10 (9–11)	12 (11–13)	<0.001
**Length of ICU stay, days**	6 (3–17)	16 (9–23)	<0.001	6 (3–14)	15 (8–24)	<0.001
28-day mortality n/total (%)	7/40 (18%)	43/74 (58%)	<0.001	4/29 (14%)	46/85 (54%)	<0.001
3-month mortality n/total (%)	10/40 (25%)	59/74 (80%)	<0.001	6/29 (21%)	63/85 (74%)	<0.001

BMI: Body mass index; ICU: Intensive care unit; APACHE: Acute physiology and chronic health evaluation; SOFA: Sequential organ failure assessment; MELD: Model of end-stage liver disease.

**Table 4:** Correlations analyses of NUTRIC and mNUTRIC with MELD, CHILD, interleukin 6, baseline albumin and body mass index (BMI) on admission to ICU.

		Spearman’s Coefficient r_s_	Linear Regression R^2^	*p*-Value
MELD	NUTRIC	0.492	0.247	<0.001
	mNUTRIC	0.475	0.224	<0.001
CHILD	NUTRIC	0.441	0.203	<0.001
	mNUTRIC	0.413	0.180	<0.001
IL6	NUTRIC	0.574	0.034	<0.001
	mNUTRIC	0.446	0.021	<0.001
albumin	NUTRIC	−0.249	0.061	0.010
	mNUTRIC	−0.232	0.052	0.013
BMI	NUTRIC	0.067	0.005	0.479
	mNUTRIC	0.076	0.006	0.422

MELD: Model of end-stage liver disease; BMI: Body mass index; ICU: Intensive care unit; IL6: Interleukin 6.

## Data Availability

More detailed data are available upon request. To receive anonymized data readers are welcome to contact the corresponding author: Ulrich Mayr, Klinik und Poliklinik für Innere Medizin II, Klinikum rechts der Isar der Technischen Universität München, Ismaninger Strasse 22, D-81675 München, Germany. Fax: 0049-89-4140-4742. E-mail: mayr.ulrich@gmx.net.

## Supplementary Materials

The following are available online at https://www.mdpi.com/2072-6643/12/7/2134/s1, Table S1: Comparison of baseline characteristics from patients ‘Included’ (*n* = 114) and patients ‘Not included’ (*n* = 36) in this study; Table S2: Univariate analyses of various baseline parameters to predict 28-day and 3-month mortalities; Table S3: Multivariate analyses to predict 28-day and 3-month mortalities; Figure S1: Prognostic accuracy of baseline NUTRIC and mNUTRIC to predict indication for hemodialysis therapy during ICU stay.

## Abbreviations

ICU	Intensive care unit
NUTRIC	Nutrition Risk in Critically ill
APACHE	Acute and physiology chronic health evaluation
SOFA	Sequential organ failure assessment
IL6	Interleukin 6
mNUTRIC	Modified Nutrition Risk in Critically ill
MELD	Model of end-stage liver disease
ECLIA	Electrochemiluminescence immunoassay
IQR	Interquartile range
ROC	Receiver-operating-characteristic curves
AUC	Area under curve
PPV	Positive predictive value
NPV	Negative predictive value
HR	Hazard ratio
BMI	Body mass index
NAFLD	Non-alcoholic fatty liver disease
HRS	Hepato-renal syndrome
CI	Confidence interval
CAID	Cirrhosis associated immune dysfunction
CRP	C-reactive protein
PCT	Procalcitonin
