# Supplementary material for: NUTRIC and Modified NUTRIC are Accurate Predictors of Outcome in End-Stage Liver Disease: A Validation in Critically Ill Patients with Liver Cirrhosis
Source: Nutrients. 2020 Jul 17;12(7):2134. doi: 10.3390/nu12072134 (PMC7400844; doi:10.3390/nu12072134)

Table S1: Comparison of baseline	´Included´ patients N=114	´Not included´ patients N=36	p-value
**Male sex, n/total (%)**	72/114 (63 %)	26/36 (72 %)	
**Age, years**	61 (52-67)	59 (48-70)	0.889
**Body weight, kg**	75 (68-85)	70 (63-82)	0.157
**Body height, cm**	175 (167-180)	174 (168-180)	0.808
**BMI, kg/m²**	24.8 (22.5-27.7)	23.2 (21.4-26.3)	0.079
**APACHE II**	22 (17-28)	22 (16-28)	0.850
**SOFA**	10 (8-13)	9 (7-15)	0.887
**MELD**	26 (22-32)	26 (23-31)	0.928
**Child-Pugh**	11 (10-13)	11 (10-13)	0.488
**Child C, n/total (%)**	98/114 (86 %)	29/36 (81 %)	
**Co-morbidities**	3 (2-3)	3 (2-3)	0.470
**Days from hospital to ICU**	2 (0-5)	3 (1-5)	0.672
**mNUTRIC**	6 (4-7)	6 (4-7)	0.375
**Etiology of cirrhosis, n/total (%)**	Alcoholic 78/114 (68 %)<br>Viral 9/114 (8 %)<br>Autoimmune 5/114 (4 %)<br>Cryptogenic/NAFLD 22/114 (20 %)	Alcoholic 25/36 (69 %)<br>Viral 4/36 (11 %)<br>Autoimmune 1/36 (3 %)<br>Cryptogenic/NAFLD 6/36 (17 %)	
**Admission diagnoses, n/total (%)**	Sepsis/Pneumonia 50/114 (44 %)<br>Acute kidney failure/HRS 24/114 (21 %)<br>Gastrointestinal bleeding 20/114 (18 %)<br>Encephalopathy/delirium 20/114 (17 %)	Sepsis/Pneumonia 12/36 (33 %)<br>Acute kidney failure/HRS 6/36 (17 %)<br>Gastrointestinal bleeding 12/36 (33 %)<br>Encephalopathy/delirium 6/36 (17 %)	
**Length of ICU-stay, days**	13 (6-22)	12 (6-25)	0.723
**28-days mortality, n/total (%)**	50/114 (44 %)	15/36 (42 %)	

**3-months mortality, n/total (%)**	69/114 (61 %)	22/36 (61 %)	0.871
**Clinical cause of death, n/total (%)**	Sepsis, Pneumonia 41/69 (61 %)<br>Cardiocirculatory failure 13/69 (19 %)<br>Gastrointestinal bleeding 11/69 (16 %)<br>Central-nervous limitations 3/69 (4 %)	Sepsis, Pneumonia 10/22 (45 %)<br>Cardiocirculatory failure 5/22 (23 %)<br>Gastrointestinal bleeding 5/22 (23 %)<br>Central-nervous limitations 2/22 (9 %)	
**Baseline creatinine, mg/dL**	1.8 (1.2-2.7)	1.7 (1.2-2.6)	0.572
**Dialysis before ICU, n/total (%)**	2/114 (1.8 %)	2/36 (5.6 %)	
**Dialysis during ICU, n/total (%)**	66/112 (59 %)	18/36 (50 %)	

BMI: Body mass index

APACHE: Acute physiology and chronic health evaluation

SOFA: Sequential organ failure assessment

MELD: Model of end-stage liver disease

ICU: Intensive care unit

NAFLD: Non-alcoholic fatty liver disease

HRS: Hepato-renal syndrome

Table S2 : Univariate analyses of various baseline parameters to predict 28-days mortality and 3-months mortality

	28-days mortality			3-months mortality		
**Univariate logistic regression**	**Beta-coefficient**	**Standard error**	**p-value**	**Beta-coefficient**	**Standard error**	**p-value**
**Male sex**	-0.241	0.391	0.537	-0.251	0.401	0.531
**Age**	0.017	0.016	0.274	0.031	0.016	0.053
**Height**	0.005	0.245	0.830	-0.006	0.025	0.824
**Weight**	-0.003	0.012	0.804	0.002	0.013	0.905
**BMI**	-0.024	0.046	0.605	0.013	0.046	0.784
**Albumin**	-0.153	0.251	0.544	-0.384	0.258	0.137
**Co-morbidities**	0.620	0.217	**0.004**	0.834	0.239	**<0.001**
**Days from hospital to ICU**	0.066	0.042	0.112	0.049	0.044	0.261
**Interleukin 6**	0.0004	0.0002	0.073	0.0003	0.0002	0.173
**Creatinine**	0.247	0.127	0.052	0.397	0.164	**0.016**
**APACHE**	0.162	0.037	**<0.001**	0.203	0.043	**<0.001**
**SOFA**	0.264	0.060	**<0.001**	0.335	0.074	**<0.001**
**MELD**	0.161	0.036	**<0.001**	0.178	0.039	**<0.001**
**CHILD**	0.550	0.138	**<0.001**	0.574	0.142	**<0.001**
**NUTRIC**	0.680	0.139	**<0.001**	0.791	0.151	**<0.001**
**mNUTRIC**	0.616	0.143	**<0.001**	0.773	0.151	**<0.001**

BMI:	Body mass index
ICU:	Intensive care unit
APACHE:	Acute physiology and chronic health evaluation
SOFA:	Sequential organ failure assessment
MELD:	Model of end-stage liver disease
NUTRIC:	Nutrition Risk in Critically ill
mNUTRIC:	Modified Nutrition Risk in Critically ill

Table S3 : Multivariate analyses to predict 28-days mortality and 3-months mortality

	28-days mortality			3-months mortality		
**Multivariate logistic regression**	**Beta-coefficient**	**Standard error**	**p-value**	**Beta-coefficient**	**Standard error**	**p-value**
**Model 1**						
**NUTRIC**	0.668	0.268	**0.013**	0.783	0.319	**0.014**
**APACHE II**	-0.053	0.082	0.513	0.018	0.092	0.841
**SOFA**	0.001	0.111	0.990	-0.115	0.133	0.386
**MELD**	0.099	0.049	**0.045**	0.174	0.073	**0.016**
**CHILD**	0.124	0.199	0.533	-0.003	0.241	0.990
**Co-morbidities**	0.080	0.281	0.776	0.248	0.308	0.421
**Creatinine**				-0.290	0.210	0.166
**Model 1**						
**mNUTRIC**	0.518	0.293	0.077	0.528	0.341	0.122
**APACHE II**	-0.023	0.083	0.776	0.063	0.092	0.497
**SOFA**	0.025	0.109	0.816	-0.068	0.130	0.601
**MELD**	0.094	0.048	0.051	0.160	0.069	**0.021**
**CHILD**	0.157	0.196	0.422	0.058	0.233	0.805
**Co-morbidities**	0.123	0.289	0.672	0.362	0.311	0.245
**Creatinine**				-0.268	0.207	0.197

**Model 2**						
**NUTRIC**	0.552	0.149	**<0.001**	0.724	0.170	**<0.001**
**MELD**	0.093	0.047	**0.048**	0.154	0.068	**0.025**
**CHILD**	0.110	0.196	0.574	-0.004	0.233	0.987
**Creatinine**				-0.231	0.203	0.252
**Model 2**						
**mNUTRIC**	0.532	0.153	**0.001**	0.690	0.171	**<0.001**
**MELD**	0.095	0.046	**0.039**	0.149	0.065	**0.022**
**CHILD**	0.141	0.193	0.466	0.045	0.223	0.840
**Creatinine**				-0.217	0.198	0.273

NUTRIC: Nutrition Risk in Critically ill

mNUTRIC: Modified Nutrition Risk in Critically ill

APACHE: Acute physiology and chronic health evaluation

SOFA: Sequential organ failure assessment

MELD: Model of end-stage liver disease

Figure S1: Prognostic accuracy of baseline NUTRIC and mNUTRIC to predict indication for hemodialysis therapy during ICU-stay

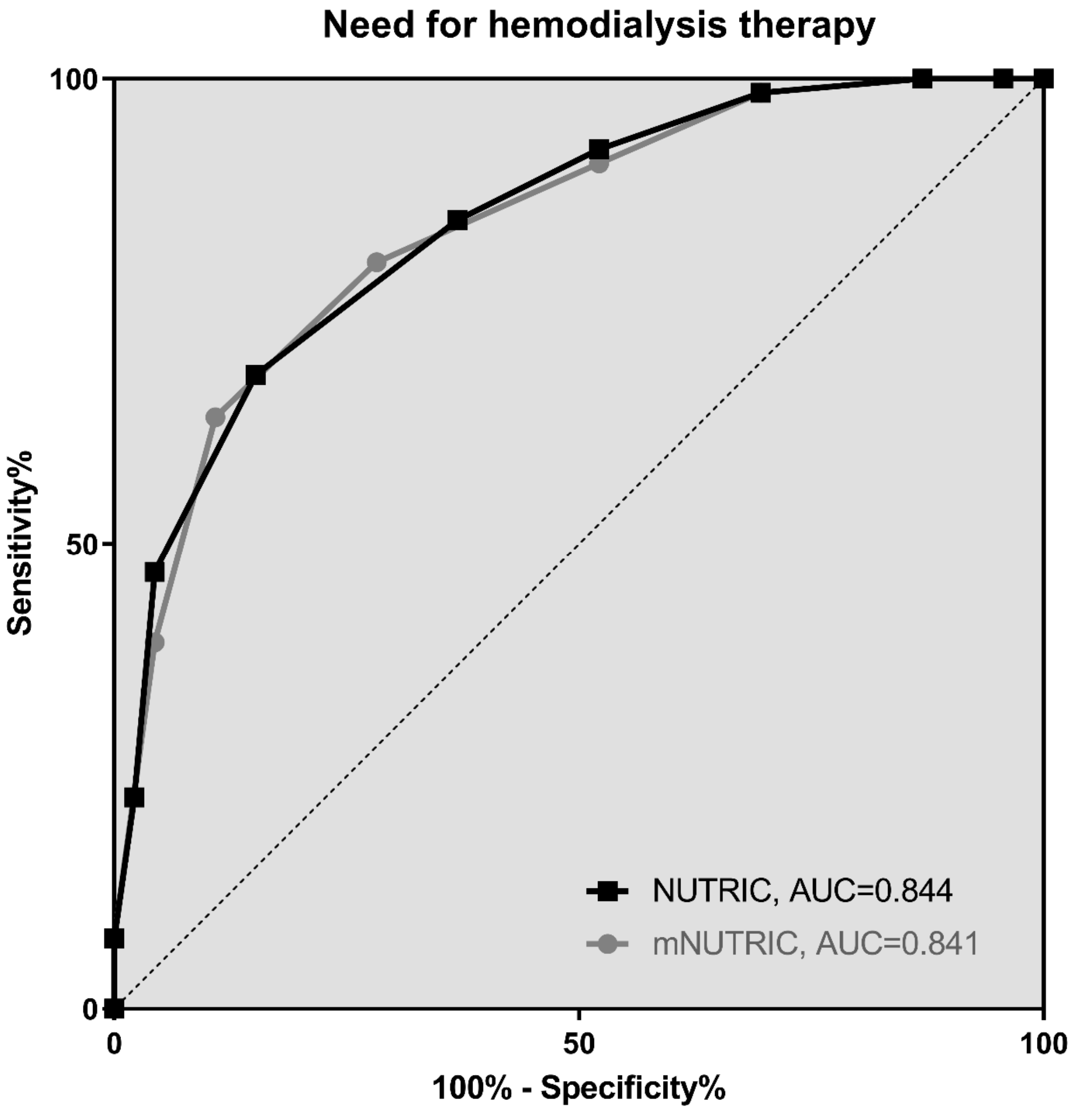

Supplement: Supplementary file 1 [file nutrients-12-02134-s001.pdf]
